# The Performance of ChatGPT-4o and DeepSeek-R1 in Interpreting Thyroid Nodule Ultrasound Text Reports: Multicenter Study

**DOI:** 10.2196/93890

**Published:** 2026-07-28

**Authors:** Yujie Xie, Jiarui Liu, Bing Zhan, Kangfan Zhang, Yuchen Li, Chunping Ning

**Affiliations:** 1 Department of Ultrasound, Affiliated Hospital of Qingdao University, No. 16 Jiangsu Road, Qingdao, Shandong, 266000, China, 86 18661806751; 2 Department of Ultrasound, JiaoZhou Central Hospital of Qingdao, Qingdao, China; 3 Department of Ultrasound, Tai'an City Central Hospital, Tai'an, China

**Keywords:** large language model, artificial intelligence, ChatGPT, DeepSeek, thyroid nodule, ultrasound, Chinese Thyroid Imaging Reporting and Data System, C-TIRADS

## Abstract

**Background:**

Although thyroid nodules are detected in up to 60% of adults on ultrasound, the vast majority are benign, creating a substantial decision-making burden compounded by heterogeneous practice guidelines. Large language models (LLMs) show promise in processing unstructured medical text and are emerging as tools for report interpretation among both clinicians and patients. However, their reliability across distinct clinical tasks in thyroid ultrasound interpretation remains poorly characterized.

**Objective:**

This study evaluates 2 LLMs, ChatGPT-4o and DeepSeek-R1, in interpreting thyroid nodule ultrasound text reports across three clinical tasks—benign-malignant differentiation, Chinese Thyroid Imaging Reporting and Data System (C-TIRADS) classification, and management recommendation—with concurrent assessment of output stability for each task.

**Methods:**

We retrospectively analyzed 1063 ultrasound text reports from 3 medical centers, including 306 with histopathological confirmation. Each nodule report was submitted to both LLMs via their consumer web interfaces using task-specific prompts, with 5 repetitions per model; final outputs were determined by mode voting. Diagnostic performance was assessed by receiver operating characteristic analysis with DeLong testing; agreement was quantified using squared weighted κ and Cohen κ; and stability was measured using Krippendorff α and Fleiss κ.

**Results:**

For benign-malignant differentiation, DeepSeek-R1 showed higher sensitivity (0.879 vs 0.692; *P*<.001) and accuracy (0.729 vs 0.644; *P*=.008) than ChatGPT-4o. With access to images and clinical context unavailable to the LLMs, senior radiologists showed higher performance (area under the curve=0.865; accuracy=0.804). For C-TIRADS classification, DeepSeek-R1 showed substantial agreement with radiologists, exceeding ChatGPT-4o (κ=0.770 vs 0.688; Δκ=0.082, 95% CI 0.048-0.122). Both models yielded moderate, comparable agreement with clinicians on management recommendations (κ=0.606 vs 0.608). Stability was near perfect for C-TIRADS classification (α=0.864 vs 0.866) and management recommendations (κ=0.853 vs. 0.849) in both models; however, DeepSeek-R1 showed markedly greater stability than ChatGPT-4o in benign-malignant differentiation (κ=0.869 vs 0.609; Δκ=0.260, 95% CI 0.191-0.321).

**Conclusions:**

Both LLMs demonstrate clinical potential for thyroid nodule ultrasound report interpretation, with DeepSeek-R1 showing advantages in diagnostic accuracy, classification consistency, and output stability. However, both LLMs remained inferior to senior radiologists, suggesting their role as decision-support tools rather than stand-alone diagnostic systems. These findings provide preliminary evidence to inform the responsible integration of LLMs into thyroid imaging workflows while highlighting the need for further evaluation before patient-facing deployment.

## Introduction

Thyroid nodules are among the most common endocrine findings in adults, detected in up to 60% of healthy individuals on ultrasound screening [1-3]. However, 90% to 95% are histologically benign, and most malignancies follow an indolent clinical course [4]. This combination of a high detection rate and a low malignancy risk imposes a substantial decision-making burden on clinicians, further compounded by considerable heterogeneity in diagnostic and management practices across guidelines, regions, and institutions [5,6]. Notable variability persists in key clinical decisions, including surveillance intervals, indications for fine-needle aspiration (FNA), and the timing of intervention [7,8].

Recent advances in AI—particularly large language models (LLMs)—have introduced a transformative approach for processing and interpreting unstructured medical data [9,10]. Emerging evidence indicates that LLMs such as GPT-3.5 (OpenAI), GPT-4.0, and Microsoft Bing can automate the structured processing of imaging reports [11-13], extract diagnostic information from free-text content [14], and respond to patient-facing medical queries [15]. In thyroid imaging specifically, recent work has explored multimodal LLMs for ultrasound-based nodule classification [16]. However, the role of LLMs in interpreting text-based thyroid ultrasound reports remains poorly defined, with their performance across different clinical tasks and the reliability of their outputs yet to be systematically characterized.

Against this backdrop, LLMs could play a meaningful role in 3 distinct scenarios. The first is structured interpretation, in which LLMs assign standardized risk categories such as Chinese Thyroid Imaging Reporting and Data System (C-TIRADS) based on textual sonographic descriptions. The second is clinical decision support, in which LLMs generate management recommendations to assist clinicians. The third is text-based diagnostic assessment, in which LLMs independently infer benign-malignant likelihood from report content—a scenario of particular relevance as patients increasingly consult publicly accessible LLMs to interpret their own reports. Across all 3 scenarios, output stability remains a foundational prerequisite, given the inherent stochasticity of generative models.

In light of these gaps, we conducted a multicenter study evaluating 2 leading LLMs—ChatGPT-4o and DeepSeek-R1—across 3 corresponding tasks: (1) benign-malignant differentiation, (2) C-TIRADS classification, and (3) management recommendation, with concurrent assessment of output stability across repeated queries. By benchmarking LLM performance across these clinically meaningful dimensions, our findings aim to inform the professional use of LLMs in thyroid nodule ultrasound interpretation, with direct relevance to emerging patient-facing applications and to support the evidence-based integration of generative AI into clinical practice.

## Methods

### Ethical Considerations

**Figure 1.**
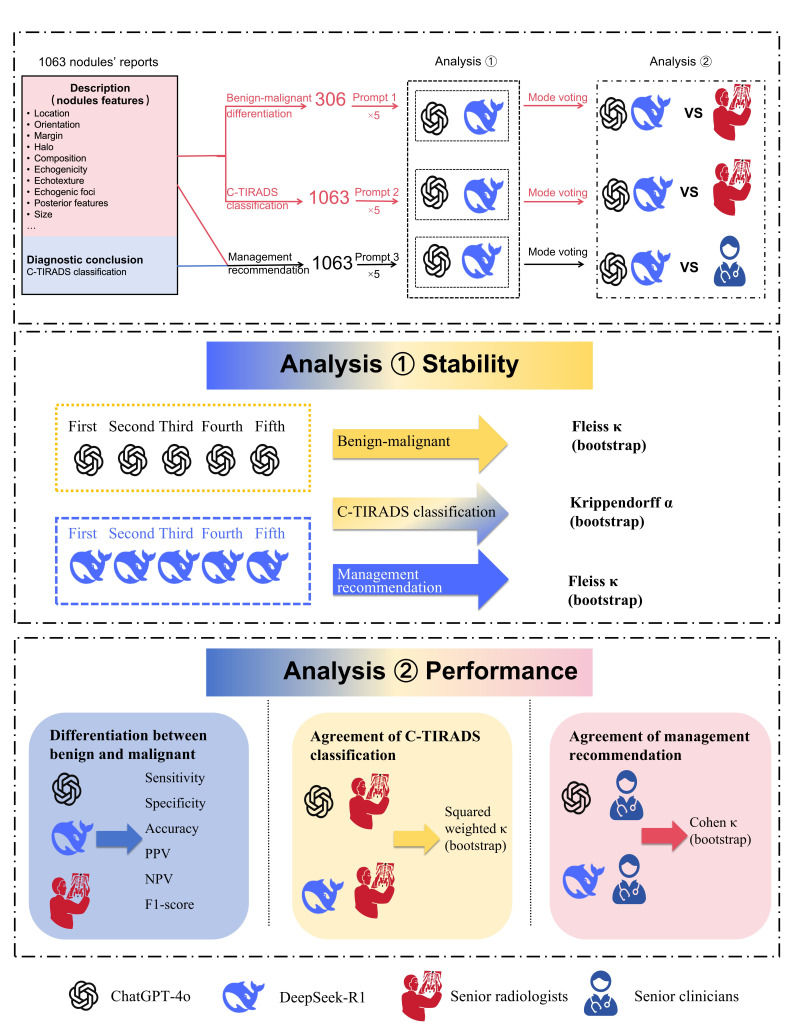
Overall study workflow. ChatGPT-4o and DeepSeek-R1 were evaluated on thyroid nodule ultrasound text reports for benign-malignant differentiation, C-TIRADS classification, and management recommendation. For each task, each model was queried five times per report using a task-specific prompt. Repeated outputs were used for 2 downstream analyses: stability assessment (middle panel) and task-specific evaluation based on the mode-voted final result (lower panel), with stability presented before performance in the revised workflow. C-TIRADS: Chinese Thyroid Imaging Reporting and Data System; LLMs: large language models; NPV: negative predictive value; PPV: positive predictive value.

This multicenter study was conducted in accordance with the Declaration of Helsinki and received approval from the ethics committee (QYFY WZLL 30331). The requirement for informed consent was waived due to the retrospective study design. Data input into LLMs were deidentified. No identifiable participant images are included in this manuscript or its supplementary materials. The overall study design is illustrated in Figure 1.

### Data

Ultrasound text reports of 1063 thyroid nodules were collected from 3 medical centers (A, B, and C). Center-level characteristics, including ultrasound equipment, the qualifications of radiologists and clinicians, and report templates, are summarized in Table S1 in Multimedia Appendix 1, while cross-center variations in reporting language are documented in Table S2 in Multimedia Appendix 1. The cases were collected between January 2023 and December 2024. Of these, 306 nodules had definitive cytopathological or histopathological diagnoses. Inclusion criteria were as follows: (1) thyroid nodules examined by senior radiologists; (2) a single nodule or, in cases with multiple nodules, the most representative nodule, defined as the one with the highest C-TIRADS category—if multiple nodules shared the same category, the largest nodule (by maximum diameter) was selected; and (3) a complete histological report for pathologically diagnosed cases. Exclusion criteria were as follows: (1) patients with other thyroid diseases; (2) incomplete sonographic descriptions of nodule characteristics or the radiologist’s diagnostic conclusion; (3) unclear management recommendations; and (4) pathological results lacking a definitive benign or malignant classification, including cytological results corresponding to Bethesda category I (nondiagnostic), III (atypia of undetermined significance), IV (follicular neoplasm), or V (suspicious for malignancy), and histopathological reports with indeterminate diagnoses (eg, tumors of uncertain malignant potential).

Histopathological confirmation was available only for patients who underwent FNA or thyroidectomy based on clinical indications (eg, C-TIRADS ≥4a, progressive enlargement, or patient preference), which inherently enriched this subset with higher-risk nodules.

### LLMS

Two LLMs, ChatGPT-4o and DeepSeek-R1 (671B parameters; Mixture-of-Experts architecture), were employed in this study. DeepSeek-R1 was used with the “Deep Think” mode enabled. Neither model underwent additional training on dedicated medical imaging datasets.

### Standardized Prompt

Consistent with clinical practice, all prompts were developed and administered in Chinese, with original ultrasound reports input directly without translation. A zero-shot prompting approach was adopted for all tasks. Each prompt constrained the output to a predefined categorical format. English translations, verified by 2 bilingual medical researchers, are provided for reference only (Table S3 in Multimedia Appendix 1).

### Query Execution

All queries were performed manually by 3 trained operators from May 3 to June 27, 2025, during which no core model updates were released for either LLM. Both models were accessed through their official consumer-facing web interfaces (chat.openai.com and chat.deepseek.com); no browser-based automation, API calls, or third-party plugins were used. To ensure independence across successive queries, all operators followed a standardized operating procedure incorporating three safeguards: (1) each query was initiated in a newly opened chat session, (2) the conversation was manually deleted from the chat history immediately after response recording, and (3) no user-specific personalization features (eg, custom instructions or persistent memory) were enabled on either platform. Workload was evenly distributed across the 3 operators to minimize operator-specific bias.

### Experimental Program

#### Benign-Malignant Differentiation

Sonographic descriptions of 306 pathologically confirmed thyroid nodules were independently input into 2 LLMs five times each using standardized prompt 1. Given the inherent stochasticity of LLM outputs, a five-repetition design with mode voting was adopted to generate a stable consensus prediction, consistent with prior LLM-based medical studies [17-19]. The consensus diagnosis was compared with histopathology as the gold standard. Model stability was evaluated by comparing the 5 individual outputs for each LLM.

#### C-TIRADS Classification

Sonographic descriptions were input into 2 LLMs five times each using standardized prompt 2. The final C-TIRADS category was determined by mode voting (as described above). When multiple modes were present, a tie-breaking rule was applied in which the highest category was selected to prioritize patient safety by minimizing the risk of underclassification. Agreement was gaged by comparing the ultimate classification with the senior radiologist’s assessment, while stability was evaluated by examining the 5 outputs generated by each LLM.

#### Management Recommendation

To simulate the real-world clinical workflow, the comprehensive report, including both the sonographic descriptions and the radiologist’s C-TIRADS conclusion, was input into 2 LLMs five times each using standardized prompt 3. The output space was restricted to a binary choice (follow-up vs FNA), consistent with the initial ultrasound-based decision node defined in major thyroid nodule guidelines [20-22]; outputs recommending surgical referral were classified as format errors (Table S4 in Multimedia Appendix 1). The final recommendation was determined using mode voting (as described above) and assessed for consistency with the clinician’s assessment. The stability of each LLM’s 5 recommendations was evaluated through comparative analysis.

For benign-malignant differentiation, histopathological diagnosis served as the gold standard. For C-TIRADS classification, which lacks an objective ground truth, senior radiologists’ original assessments at each center served as the reference, with interrater variability minimized through the unified 2020 C-TIRADS guidelines and structured reporting templates (Table S1 in Multimedia Appendix 1). For management recommendations, the documented decisions of attending clinicians served as the reference. Accordingly, performance on the latter 2 tasks should be interpreted as agreement with expert raters rather than diagnostic accuracy. Notably, LLMs were evaluated using text reports alone, whereas radiologists and clinicians had full access to ultrasound images and patient clinical information.

### Nonstandard Output

All LLM outputs were independently reviewed by 2 investigators to identify nonstandard outputs, defined as any response that could not be directly mapped to a predefined answer category. Nonstandard outputs were classified into 3 mutually exclusive categories: format errors, equivocal responses, and hallucinations. Disagreements were resolved by consensus with a third investigator. Operational definitions and representative examples are provided in Table S4 in Multimedia Appendix 1.

### Statistical Analyses

Baseline comparability across the three centers was assessed using one-way ANOVA for age, the chi-square test for sex, and the Kruskal-Wallis test for C-TIRADS distribution. For the pathology-confirmed subset, between-center comparisons were performed using the Wilcoxon rank-sum test for age and C-TIRADS distribution, and the chi-square test for sex and malignancy proportion.

Diagnostic performance was evaluated using receiver operating characteristic (ROC) curve analysis with the area under the curve (AUC). For the senior radiologists, C-TIRADS categories served as the ordinal predictor for ROC construction. The consumer-facing web interfaces of ChatGPT-4o and DeepSeek-R1 do not expose native token-level probabilities [23], precluding direct probabilistic ROC analysis. To ensure a methodologically equivalent comparison, the count of malignant verdicts (range: 0‐5) across 5 independent evaluation sessions served as the ordinal predictor of malignancy for the LLMs, in line with the self-consistency paradigm [24], in which aggregating multiple independent inferences yields more reliable predictions than any single run [24,25]. Paired AUC comparisons were performed using the DeLong test on the same set of 306 pathologically confirmed nodules.

Sensitivity, specificity, positive predictive value (PPV), negative predictive value (NPV), accuracy, and *F*_1_-score were calculated from mode-voting binary predictions for the LLMs. For the senior radiologists, the optimal binarization threshold was determined by maximizing the Youden index from the ROC curve, and the same metrics were calculated accordingly. Between-model comparisons of sensitivity, specificity, and accuracy were conducted using the McNemar test with continuity correction. For PPV, NPV, and *F*_1_-score, the differences between the 2 models and their 95% CI were estimated using the bias-corrected and accelerated bootstrap method as described below.

The agreement of C-TIRADS classification between LLMs and radiologists was assessed using the squared weighted κ coefficient [26]; Cohen κ coefficient was employed to evaluate agreement in management recommendations between LLMs and clinicians. To evaluate the robustness of the C-TIRADS classification results to the predefined higher-category tie-breaking rule, a sensitivity analysis was performed by alternatively assigning tied cases to the lower category, and the squared weighted κ with senior radiologists was recalculated under both strategies.

Krippendorff α coefficient was used to assess the internal stability of C-TIRADS classification across repeated runs of the 2 LLMs. The stability of management recommendations and benign-malignant classification was evaluated using Fleiss κ coefficient.

The choice of these agreement and stability indices followed standard practice: squared weighted κ [26] penalizes larger category discrepancies more heavily than smaller ones, thereby accounting for ordinal proximity in C-TIRADS categories; Cohen κ is appropriate for binary agreement; Krippendorff α handles multicategory stability with multiple measurements; and Fleiss κ extends to binary stability across repeated runs.

All 95% CIs for point estimates were calculated using the bias-corrected and accelerated bootstrap method with 5000 resamples (case-level resampling with replacement). For between-LLM comparisons of agreement or stability metrics, the 95% CI for the difference (DeepSeek-R1 − ChatGPT-4o) was computed using the same procedure. For the tie-breaking sensitivity analysis, the between-strategy difference in weighted κ was defined as Δκ=κ_high_ − κ_low_. Differences were considered statistically significant when the CI excluded zero.

### Statistical Software and Interpretation Criteria

All statistical analyses were conducted using R software (version 4.4.3; R Foundation for Statistical Computing), with 2-sided *P*<.05 considered statistically significant. The following thresholds were used to interpret κ and α values, according to the criteria proposed by Landis and Koch [27]: ≤0.20, poor; 0.21‐0.40, fair; 0.41‐0.60, moderate; 0.61‐0.80, substantial; and 0.81‐1.00, almost perfect agreement.

## Results

### Patient and Center Characteristics

A total of 1063 thyroid nodules from 3 centers—Center A (n=448), Center B (n=307), and Center C (n=308)—were included in this study. The cohort comprised 740 (69.6%) female and 323 (30.4%) male patients, with a mean age of 46.59 (SD 14.46) years (range: 4‐90 y). Baseline comparisons across centers revealed significant differences in age and C-TIRADS distribution (*P*<.001), whereas sex distribution was comparable (*P*=.30; Table 1).

**Table 1.** Demographic and clinical characteristics of the study population^a^.

Characteristic	Overall (N=1063)	Center A (n=448)	Center B (n=307)	Center C (n=308)	*P* value
Age (years), mean (SD; range)	46.59 (14.46; 4-90)	43.58 (14.10; 4-85)	51.32 (14.26; 16-90)	46.26 (13.99; 11-81)	<.001
Sex, n (%)					.30
Male	323 (30.4)	135 (30.1)	85 (27.7)	103 (33.4)	
Female	740 (69.6)	313 (69.9)	222 (72.3)	205 (66.6)	
C-TIRADS^b^ category, n (%)					<.001
1	47 (4.4)	30 (6.7)	7 (2.3)	10 (3.2)	
2	115 (10.8)	49 (10.9)	29 (9.4)	37 (12.0)	
3	278 (26.2)	75 (16.7)	121 (39.4)	82 (26.6)	
4a	285 (26.8)	131 (29.2)	92 (30.0)	62 (20.1)	
4b	186 (17.5)	78 (17.4)	39 (12.7)	69 (22.4)	
4c	111 (10.4)	53 (11.8)	18 (5.9)	40 (13.0)	
5	41 (3.9)	32 (7.1)	1 (0.3)	8 (2.6)	

a Data are presented as mean (SD; range) for continuous variables and n (%) for categorical variables.

b C-TIRADS: Chinese Thyroid Imaging Reporting and Data System.

Among the overall cohort, 306 nodules (from Centers A and B; Center C did not contribute pathology-confirmed cases) had histopathological confirmation, including 124 (40.5%) benign and 182 (59.5%) malignant lesions. The pathology-confirmed subset showed a significant difference in age between the 2 centers (*P*=.002), while sex and C-TIRADS distributions were comparable (*P*=.70 and *P*=.17, respectively). The malignancy rate was significantly higher in Center B than in Center A (59/84, 70.2% vs 123/222, 55.4%; *P*=.03; Table 2).

**Table 2.** Demographic and clinical characteristics of the pathology-confirmed subset for benign-malignant differentiation analysis^a^.

Characteristic	Total (N=306)	Center A (n=222)	Center B (n=84)	*P* value
Age (y), mean (SD; range)	45.83 (13.69; 4‐85)	44.40 (14.00; 4‐85)	49.70 (12.00; 17‐81)	.002
Sex, n (%)				.70
Male	87 (28.4)	65 (29.3)	22 (26.2)	
Female	219 (71.6)	157 (70.7)	62 (73.8)	
C-TIRADS^b^ category, n (%)				.17
3	25 (8.2)	18 (8.1)	7 (8.3)	
4a	95 (31.0)	66 (29.7)	29 (34.5)	
4b	84 (27.5)	61 (27.5)	23 (27.4)	
4c	73 (23.9)	48 (21.6)	25 (29.8)	
5	29 (9.5)	29 (13.1)	0 (0.0)	
Histopathological diagnosis, n (%)				.03
Benign	124 (40.5)	99 (44.6)	25 (29.8)	
Malignant	182 (59.5)	123 (55.4)	59 (70.2)	

a Data are presented as mean (SD; range) for continuous variables and n (%) for categorical variables. Center C did not contribute pathology-confirmed cases. C-TIRADS categories 1 and 2 are absent because such nodules lacked histopathological reference standards.

b C-TIRADS: Chinese Thyroid Imaging Reporting and Data System.

### Benign-Malignant Differentiation

**Figure 2.**
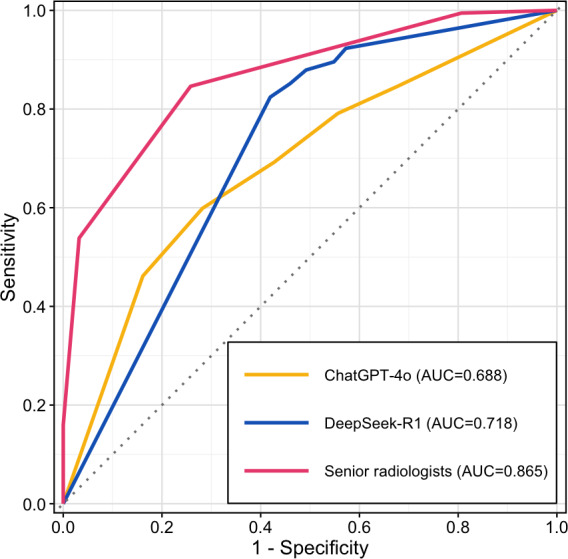
Receiver operating characteristic curves for ChatGPT-4o, DeepSeek-R1, and senior radiologists. AUC: area under the curve.

Among the 306 pathology-confirmed nodules (malignancy prevalence: n=182, 59.5%), the diagnostic performance of both LLMs was compared with that of senior radiologists. The AUC of DeepSeek-R1 was 0.718 (95% CI 0.665-0.770), numerically higher than that of ChatGPT-4o (AUC=0.688, 95% CI 0.629-0.746), although the difference was not statistically significant (*P*=.34). Both models showed significantly lower diagnostic performance than senior radiologists, who achieved an AUC of 0.865 (95% CI 0.828-0.902; both *P*<.001 vs the LLMs; Figure 2).

**Figure 3.**
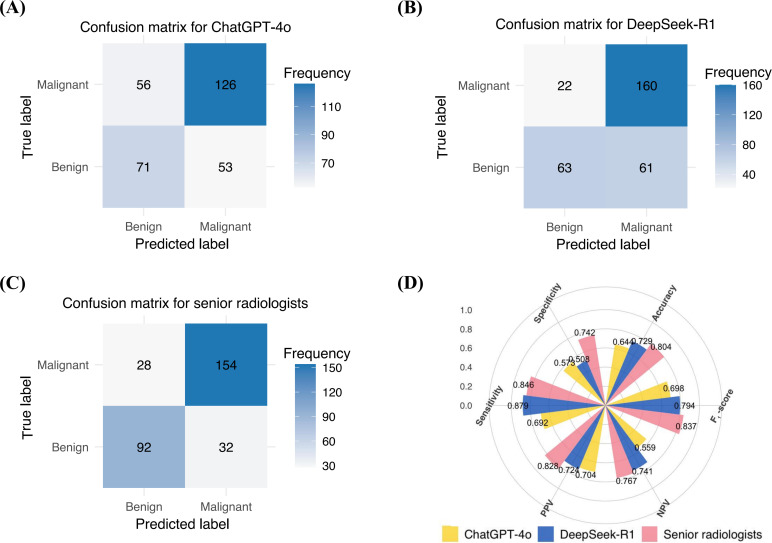
(A) Confusion matrix for ChatGPT-4o. (B) Confusion matrix for DeepSeek-R1. (C) Confusion matrix for senior radiologists. (D) Comparison of performance: ChatGPT-4o vs DeepSeek-R1 vs senior radiologists. NPV: negative predictive value; PPV: positive predictive value.

Using the optimal cutoff of C-TIRADS ≥4b (Youden index=0.588), senior radiologists achieved the highest performance across most indicators (sensitivity 0.846, specificity 0.742, accuracy 0.804, PPV 0.828, NPV 0.767, and *F*_1_-score 0.837). Compared with ChatGPT-4o, DeepSeek-R1 showed significantly higher sensitivity (0.879 vs 0.692; *P*<.001), accuracy (0.729 vs 0.644; *P*=.008), and NPV (0.741 vs 0.559; Δ=0.182, 95% CI 0.088-0.271), whereas specificity (0.508 vs 0.573) and PPV (0.724 vs 0.704) were comparable between the 2 models (Figure 3; Table S5 in Multimedia Appendix 1).

### C-TIRADS Classification

DeepSeek-R1 demonstrated substantial agreement with radiologists on C-TIRADS classification, showing significantly higher concordance than ChatGPT-4o (0.770, 95% CI 0.742-0.796 vs 0.688, 95% CI 0.644-0.724; Δκ=0.082, 95% CI 0.048-0.122). Across the 3 centers, DeepSeek-R1 demonstrated moderate to almost perfect agreement (κ range: 0.686‐0.822). In contrast, ChatGPT-4o’s agreement with radiologists varied considerably, ranging from almost perfect agreement in Center A (0.788, 95% CI 0.733-0.828) to moderate agreement in Centers B and C (κ range: 0.508‐0.594; Table 3).

**Table 3.** Agreement between large language models and radiologists on Chinese Thyroid Imaging Reporting and Data System classification^a^.

Center	Nodules, n	ChatGPT-4o weighted κ (95% CI)	DeepSeek-R1 weighted κ (95% CI)	Δ weighted κ (95% CI)
A	448	0.788 (0.733 to 0.828)	0.822 (0.780 to 0.855)	0.034 (−0.010 to 0.084)
B	307	0.508 (0.403 to 0.585)	0.686 (0.638 to 0.730)	0.178 (0.102 to 0.282)
C	308	0.594 (0.503 to 0.669)	0.744 (0.685 to 0.791)	0.150 (0.087 to 0.228)
Overall	1063	0.688 (0.644 to 0.724)	0.770 (0.742 to 0.796)	0.082 (0.048 to 0.122)

a Data are weighted κ values, with 95% CIs in parentheses. Δ weighted κ represents the mean difference in κ values (DeepSeek-R1 − ChatGPT-4o).

**Figure 4.**
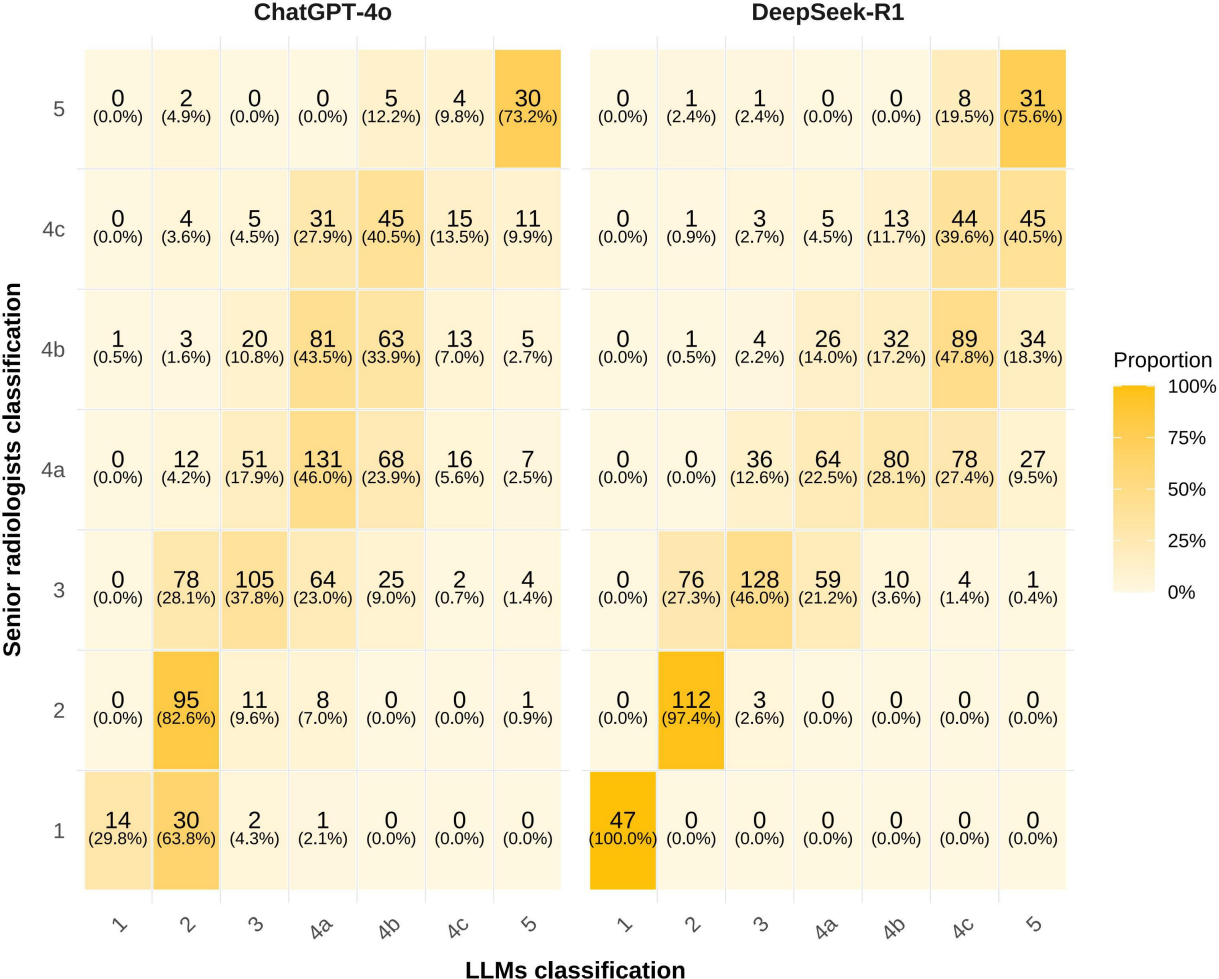
Agreement between large language models and radiologists on Chinese Thyroid Imaging Reporting and Data System classification: confusion matrix analysis. LLMs: large language models.

The classification performance was further examined in Figure 4. Both models exhibited strong agreement with radiologists for category 2 and 5 nodules (range: 73.2%‐97.4%). Challenges were observed in classifying category 4 nodules, with agreement rates ranging from 13.5% to 46.0%. For category 1 nodules, DeepSeek-R1 achieved 100% agreement, while ChatGPT-4o achieved 29.8% agreement.

Tie-breaking occurred in 5.0% (53/1063) of ChatGPT-4o classifications and 10.0% (106/1063) of DeepSeek-R1 classifications, with most events involving adjacent categories, particularly within the category 4 spectrum (Table S6 in Multimedia Appendix 1). All observed ties were 2-way; no three-or-more-way ties or fully dispersed configurations were encountered in either model. Replacing the higher-category strategy with a lower-category alternative yielded minimal changes in agreement: ChatGPT-4o (κ=0.688 vs 0.690; Δκ=−0.002, 95% CI −0.016 to 0.013) and DeepSeek-R1 (κ=0.770 vs 0.781; Δκ=−0.011, 95% CI −0.019 to −0.004), with both models maintaining substantial agreement under either strategy (Table S7 in Multimedia Appendix 1).

### Management Recommendation

**Figure 5.**
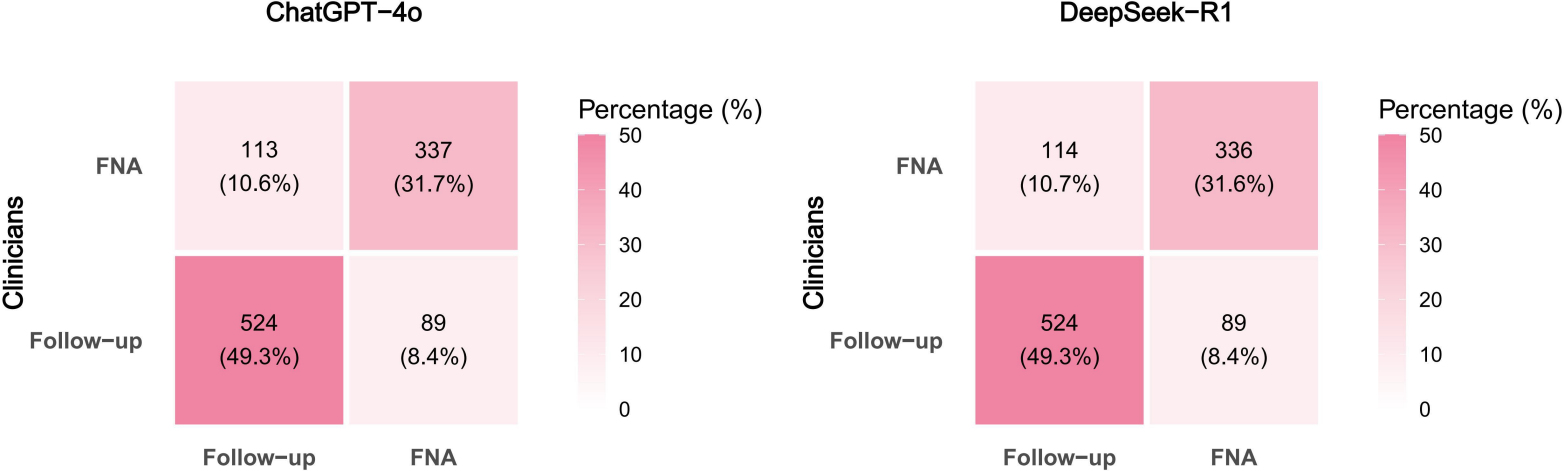
Agreement between large language models and clinicians on management recommendations: confusion matrix analysis. FNA: fine-needle aspiration.

Across the 1063 thyroid nodules, both models showed moderate and comparable agreement with clinicians (ChatGPT-4o: κ=0.608, 95% CI 0.557 to 0.654; DeepSeek-R1: κ=0.606, 95% CI 0.557 to 0.653; Δκ=−0.002, 95% CI −0.044 to 0.041). Center-level analysis showed significant differences only in Center B, where DeepSeek-R1 had higher agreement than ChatGPT-4o (κ=0.474, 95% CI 0.359 to 0.578 vs 0.342, 95% CI 0.224 to 0.462; Δκ=0.132, 95% CI 0.029 to 0.243; Table 4). The overall percentage agreement was similarly high for both models (ChatGPT-4o: 861, 81.0%; DeepSeek-R1: 860, 80.9%; Figure 5).

**Table 4.** Agreement between large language models and clinicians on management recommendations^a^.

Center	Nodules, n	ChatGPT-4o κ (95 % CI)	DeepSeek-R1 κ (95% CI)	Δ κ (95% CI)
A	448	0.642 (0.566 to 0.713)	0.590 (0.515 to 0.665)	−0.052 (−0.109 to 0.002)
B	307	0.342 (0.224 to 0.462)	0.474 (0.359 to 0.578)	0.132 (0.029 to 0.243)
C	308	0.670 (0.583 to 0.749)	0.681 (0.594 to 0.758)	0.011 (−0.064 to 0.085)
Overall	1063	0.608 (0.557 to 0.654)	0.606 (0.557 to 0.653)	−0.002 (−0.044 to 0.041)

a Data are Cohen κ values, with 95% CIs in parentheses. Δ κ represents the mean difference in κ values (DeepSeek-R1 − ChatGPT-4o).

### Output Stability

In benign-malignant differentiation, DeepSeek-R1 exhibited almost perfect stability (κ=0.869, 95% CI 0.822-0.906), significantly higher than that of ChatGPT-4o (κ=0.609, 95% CI 0.557-0.661; Δκ=0.260, 95% CI 0.191-0.321).

The overall stability of C-TIRADS classification was almost perfect and comparable between the 2 models (ChatGPT-4o: α=0.866, 95% CI 0.851 to 0.879; DeepSeek-R1: α=0.864, 95% CI 0.849 to 0.879; Δα=−0.002, 95% CI −0.023 to 0.017; Table 5). However, category-specific analysis revealed distinct patterns. For category 1 nodules, DeepSeek-R1 achieved perfect stability (α=1.000), significantly higher than ChatGPT-4o (α=0.562, 95% CI 0.395 to 0.717; Δα=0.438, 95% CI 0.297 to 0.617). Conversely, ChatGPT-4o outperformed DeepSeek-R1 in categories 3, 4a, 4b, and 4c (category 3: α=0.789, 95% CI 0.749 to 0.822 vs 0.661, 95% CI 0.608 to 0.711; Δα=−0.128, 95% CI −0.193 to −0.065]; category 4a: α=0.692, 95% CI 0.643 to 0.740 vs 0.574, 95% CI 0.517 to 0.630; Δα=−0.118, 95% CI −0.193 to −0.044; category 4b: α=0.681, 95% CI 0.610 to 0.748 vs 0.449, 95% CI 0.377 to 0.528; Δα=−0.232, 95% CI −0.334 to −0.130; category 4c: α=0.766, 95% CI 0.692 to 0.827 vs 0.490, 95% CI 0.398 to 0.597; Δα=−0.276, 95% CI −0.397 to −0.155).

**Table 5.** Stability of ChatGPT-4o and DeepSeek-R1 in Chinese Thyroid Imaging Reporting and Data System classification^a^.

Category	Nodules, n	ChatGPT-4o α (95 % CI)	DeepSeek-R1 α (95% CI)	Δα (95% CI)
Overall	1063	0.866 (0.851 to 0.879)	0.864 (0.849 to 0.879)	−0.002 (−0.023 to 0.017)
1	47	0.562 (0.395 to 0.717)	1.000 (—^b^)	0.438 (0.297 to 0.617)
2	115	0.627 (0.514 to 0.736)	0.416 (0.119 to 0.721)	−0.211 (−0.661 to 0.069)
3	278	0.789 (0.749 to 0.822)	0.661 (0.608 to 0.711)	−0.128 (−0.193 to −0.065)
4a	285	0.692 (0.643 to 0.740)	0.574 (0.517 to 0.630)	−0.118 (−0.193 to −0.044)
4b	186	0.681 (0.610 to 0.748)	0.449 (0.377 to 0.528)	−0.232 (−0.334 to −0.130)
4c	111	0.766 (0.692 to 0.827)	0.490 (0.398 to 0.597)	−0.276 (−0.397 to −0.155)
5	41	0.460 (0.282 to 0.668)	0.446 (0.275 to 0.671)	−0.014 (−0.287 to 0.277)

a Data are Krippendorff α values, with 95% CIs in parentheses. Δα represents the mean difference in α values (DeepSeek-R1 − ChatGPT-4o).

b Not applicable.

Both models demonstrated almost perfect and comparable stability in generating management recommendations (ChatGPT-4o: κ=0.849, 95% CI 0.826 to 0.870; DeepSeek-R1: κ=0.853, 95% CI 0.830 to 0.874; Δκ=0.004, 95% CI −0.026 to 0.033).

### Nonstandard Output

Of the 12,160 responses generated by each model, nonstandard outputs accounted for 169 (1.39%) in ChatGPT-4o and 80 (0.66%) in DeepSeek-R1. Hallucinations, the category most directly relevant to patient safety, accounted for 81 (0.67%) and 35 (0.29%) of outputs in ChatGPT-4o and DeepSeek-R1, respectively. Across both models, nonstandard outputs were most frequent in the benign-malignant differentiation task and least frequent in the management recommendation task. Detailed distributions by category and task are provided in Table S8 in Multimedia Appendix 1.

## Discussion

### Principal Findings

In this multicenter study, we systematically evaluated 2 prominent LLMs, DeepSeek-R1 and ChatGPT-4o, in interpreting thyroid nodule ultrasound text reports across 3 clinically relevant tasks—benign-malignant differentiation, C-TIRADS classification, and management recommendation—with concurrent assessment of output stability. Our key findings include (1) DeepSeek-R1 demonstrated higher sensitivity (0.879 vs 0.692; *P*<.001) and accuracy (0.729 vs 0.644; *P*=.008) than ChatGPT-4o in benign-malignant differentiation, though both remained inferior to senior radiologists (AUC=0.865; accuracy=0.804), (2) DeepSeek-R1 showed substantially higher agreement with radiologists in C-TIRADS classification (κ=0.770 vs 0.688; Δκ=0.082, 95% CI 0.048 to 0.122), (3) both models achieved moderate and comparable agreement with clinicians on management recommendations (κ=0.606 vs 0.608), and (4) while both models demonstrated near-perfect stability for C-TIRADS classification (α=0.864 vs 0.866) and management recommendations (κ=0.853 vs 0.849), DeepSeek-R1 exhibited markedly greater stability than ChatGPT-4o in benign-malignant differentiation (κ=0.869 vs 0.609; Δκ=0.260, 95% CI 0.191 to 0.321). These findings provide empirical evidence for the responsible integration of LLMs into thyroid imaging workflows while highlighting task-dependent performance limitations.

### Task-Specific Performance and Comparison

#### Interpretive Considerations

A conceptual distinction warrants emphasis before interpreting task-specific performance. For benign-malignant differentiation, histopathology served as the reference standard, enabling assessment of diagnostic accuracy. For C-TIRADS classification and management recommendations, the reference was human expert judgment; performance in these domains therefore reflects clinical concordance rather than objective correctness. As noted in the Introduction, considerable interclinician variability exists in thyroid nodule diagnosis and management. High agreement with expert raters indicates the model’s capacity to replicate specific practice patterns but does not inherently validate clinical correctness, as human assessments remain fallible. Ultimate validation must rest on pathological outcomes and long-term patient prognosis rather than expert consensus alone.

#### Benign-Malignant Differentiation

In the pathology-confirmed subset (n=306), DeepSeek-R1 demonstrated higher sensitivity (0.879 vs 0.692; *P*<.001) and accuracy (0.729 vs 0.644; *P*=.008) than ChatGPT-4o, though the AUC difference was not statistically significant (0.718 vs 0.688; *P*=.34). With both models approaching the conventionally acceptable diagnostic range (AUC=0.7‐0.8) [28], these statistically detectable differences should not be conflated with clinically meaningful superiority [29]. Senior radiologists achieved higher performance (AUC=0.865); however, this comparison reflects informational asymmetry rather than inherent model inferiority, as radiologists accessed ultrasound images and clinical context, whereas LLMs operated on text reports alone. This gap suggests a potential auxiliary role for LLMs in image-limited scenarios such as remote consultation, patient self-interpretation of reports, and preliminary triage in primary care [30]. Future work should prioritize multimodal medical AI [19,31] to enable more equitable comparisons.

The malignancy rate in our pathology-confirmed subset (182/306, 59.5%) substantially exceeded the general population prevalence (5%‐10%) [22,32], reflecting spectrum bias inherent to retrospective histopathology-based studies [33]. This enrichment artificially inflates PPV and overall accuracy while potentially underestimating NPV; sensitivity, specificity, and AUC, though mathematically prevalence independent, may be indirectly affected by the narrower disease spectrum [34]. Bayesian prevalence adjustment assuming real-world malignancy rates of 5% and 10% substantially reduced PPVs (ChatGPT-4o: 70.4%→7.9%-15.3%; DeepSeek-R1: 72.4%→8.6%-16.6%), while adjusted NPVs all exceeded 94% (Table S9 in Multimedia Appendix 1). These findings suggest that both models retain strong rule-out capability in low-prevalence settings, though positive predictions require histopathological confirmation.

#### C-TIRADS Classification

To our knowledge, this study represents the first application of LLMs to C-TIRADS classification based solely on sonographic descriptions. DeepSeek-R1 exhibited substantially higher concordance with senior radiologists than ChatGPT-4o (κ=0.770 vs 0.688). This difference may relate to architectural distinctions: DeepSeek-R1 uses a Mixture-of-Experts architecture with 671 billion total parameters and 37 billion activated per token [35,36], potentially enabling task-specific specialization [37], combined with multistage reinforcement learning that may enhance structured reasoning [38]. In contrast, GPT-4o, developed as a general-purpose multimodal model [39,40], has not disclosed architectural specifics, limiting direct comparison. Moreover, the activated reasoning mode in DeepSeek-R1 may also contribute to the observed gap beyond architecture alone. These considerations remain speculative, as our black-box evaluation precluded analysis of internal mechanisms. Controlled architectural comparisons under matched inference configurations are warranted in future studies.

#### Management Recommendation

Both LLMs demonstrated high raw agreement with clinicians (approximately 81%), while κ values were moderate (κ=0.606 vs 0.608). This approximately 20-percentage-point discrepancy primarily reflects κ’s mathematical properties, which account for chance agreement and are influenced by marginal category distributions [41,42]. In datasets with imbalanced distributions—such as ours, where C-TIRADS category proportions ranged from 3.9% to 26.8%—the elevated baseline probability of chance agreement suppresses κ relative to observed concordance [42]. This well-documented statistical phenomenon does not imply inadequate performance [41,42]; rather, our moderate-to-substantial κ values (0.61‐0.77) indicate promising concordance requiring prospective validation before clinical deployment.

Performance varied across management categories. For follow-up recommendations, LLM concordance was relatively high (approximately 85.5%), whereas for invasive procedures such as FNA, agreement declined to 74.7%‐74.9%. This pattern aligns with prior findings that LLM accuracy in oncologic management decision support ranges from 50% to 70% [43], underscoring proficiency in straightforward cases but inadequacy in complex decision-making [44-47]. LLMs face challenges in high-risk decisions due to difficulty incorporating individual patient factors—age, comorbidities, clinical symptoms, and socioeconomic status—essential to clinical judgment. Furthermore, they may not account for clinician caution under uncertainty or resource variability across settings [48,49]. While proficient in standardized tasks, LLMs should serve as decision-support adjuncts rather than substitutes for physician judgment in complex treatment scenarios.

#### Output Stability

Both models demonstrated near-perfect stability for C-TIRADS classification and management recommendations, supporting their reliability in structured, rule-based tasks. However, a marked disparity emerged in benign-malignant differentiation: DeepSeek-R1 maintained high stability (κ=0.869), whereas ChatGPT-4o exhibited substantially lower consistency (κ=0.609). This instability in ChatGPT-4o is particularly concerning in patient-facing contexts, where repeated queries on identical reports could yield divergent diagnostic impressions, generating unwarranted anxiety or misplaced reassurance. These findings contrast with prior reports of high ChatGPT reproducibility in tasks such as Response Evaluation Criteria In Solid Tumours (RECIST) assessment [9], underscoring the absence of universally reliable performance across clinical contexts. The observed variability across task complexities suggests that stability should be evaluated as a task-specific property rather than a global model attribute, reinforcing the need for systematic benchmarking before clinical deployment.

### Clinical Implications

The differential performance across tasks observed in our study suggests task-specific deployment strategies for LLMs in thyroid imaging workflows. For standardized tasks such as C-TIRADS classification, LLMs—particularly DeepSeek-R1—could serve as quality assurance tools to enhance reporting consistency and assist less experienced readers in adhering to structured reporting systems. For low-risk nodules requiring surveillance, LLMs might be explored as triage-support tools in primary care settings, though their actual impact on referral patterns requires prospective evaluation. For patient-facing applications, where patients increasingly consult publicly accessible LLMs to interpret their own reports, models might provide preliminary interpretations with explicit uncertainty quantification. However, the safety and benefit of such patient-facing use require dedicated evaluation before broad deployment.

Safety considerations are integral to clinical deployment. Encouragingly, both models exhibited low hallucination rates (0.29%‐0.67%)—markedly lower than the 18% to 51% reported in less constrained clinical settings [50,51]—likely reflecting structured prompts and standardized C-TIRADS-aligned descriptors that narrowed the response space [52]. Hallucinations were nonetheless most frequent in benign-malignant differentiation, consistent with the greater inferential burden of probabilistic binary judgments. Given that LLMs may deliver erroneous content with unwarranted confidence [53], these findings support their deployment in similarly structured interpretation tasks as physician-supervised decision-support tools rather than autonomous diagnostic systems. Furthermore, regulatory frameworks for AI-assisted medical decision-making remain nascent, and institutional policies must address liability, informed consent, and clinical oversight before widespread deployment.

### Limitations

Several limitations merit consideration. First, LLMs were evaluated exclusively on Chinese-language text reports, whereas senior radiologists accessed ultrasound images and clinical context; multimodal validation is warranted for fairer comparisons. Second, histopathological confirmation was obtained only for clinically indicated nodules (306/1063, 28.8%), yielding a malignancy-enriched cohort (182/306, 59.5%) that may inflate diagnostic performance through spectrum bias; although Bayesian prevalence adjustment partially mitigates this issue, prospective validation in unselected screening populations is required. For nodules without histopathology, reference standards relied on radiologist assessment and clinical follow-up, which are imperfect. Third, prompt design may have influenced model performance. The management recommendation task incorporated the radiologist-derived C-TIRADS classification as model input; accordingly, performance on this task reflects the models’ adherence to management guidelines conditional on a human-derived classification, rather than their independent capacity to derive recommendations from the sonographic description alone. The binary output design may have penalized clinically defensible surgical referral recommendations, although such outputs were rare in our cohort (ChatGPT-4o: 21/12,160, 0.17%; DeepSeek-R1: 13/12,160, 0.11%); multitiered output schemes warrant exploration in future work. In addition, each task used a single standardized prompt without embedded guideline-based criteria; future studies should therefore explore diverse prompting strategies to isolate the autonomous reasoning capacity of LLMs. Fourth, both models were accessed through their consumer web interfaces, with DeepSeek-R1 querying the “Deep Think” reasoning mode and ChatGPT-4o using its standard chat configuration. Although this reflects common real-world end-user usage, this configuration asymmetry may have partially contributed to the observed performance differences. Reproducibility under standardized API configurations remains to be established. Fifth, the low hallucination rates observed under our constrained categorical output design should not be extrapolated to open-ended clinical queries, patient-facing free-text dialogue, or unconstrained generative tasks, where substantially higher rates have been reported [50,51]; dedicated evaluation is warranted before broader deployment. Sixth, the vote-count surrogate used for LLM ROC construction should not be interpreted as a calibrated probability of malignancy. Vote dispersion across independent runs reflects interinference agreement rather than a true probabilistic confidence estimate, and its formal correspondence with native token-level probabilities warrants validation through API-based access in future work. Finally, all reports and prompts were in Chinese; DeepSeek-R1, trained on a larger Chinese-language corpus, may have benefited from language-specific optimization [54,55], although this advantage was not uniformly observed across all tasks. Combined with the single-province recruitment and retrospective output characterization noted above, these factors limit geographic, linguistic, and real-world generalizability; prospective multiregional and multilingual validation is warranted.

### Future Directions

Several avenues warrant future investigation. First, multimodal integration combining text reports with ultrasound images could narrow the performance gap with radiologists; recent advances in vision-language models provide the technical foundation for such systems. Second, prospective validation in real-world clinical workflows is essential to assess the impact on diagnostic accuracy, workflow efficiency, and patient outcomes beyond retrospective concordance metrics. Third, ensemble methods combining multiple LLMs or hybrid architectures integrating LLMs with traditional machine learning models may enhance robustness and mitigate individual model weaknesses by leveraging the complementary strengths observed between DeepSeek-R1 and ChatGPT-4o. Fourth, prompt engineering strategies—including few-shot learning, chain-of-thought prompting, and guideline-embedded prompts—should be systematically evaluated to optimize performance across clinical tasks. Fifth, longitudinal studies tracking model performance across software updates are needed, as LLM capabilities evolve rapidly, and current findings may not generalize to future versions. Finally, health economic evaluations and studies examining the integration of LLMs into clinical workflows—including their impact on clinician workload, patient satisfaction, and health care costs—are needed to inform evidence-based policy decisions on AI adoption in thyroid imaging.

### Conclusions

Advanced LLMs, particularly DeepSeek-R1, demonstrate meaningful capability in thyroid nodule ultrasound report interpretation, with consistent C-TIRADS classification, acceptable benign-malignant differentiation, and high output stability in structured tasks. However, limitations in high-risk decision-making and diagnostic inference stability—particularly for ChatGPT-4o—indicate that LLMs are better suited as decision-support adjuncts to enhance clinician efficiency rather than autonomous diagnostic systems. These findings support the responsible integration of LLMs into thyroid imaging workflows under appropriate physician oversight. Fully realizing the clinical potential of LLMs will require multimodal integration, prospective validation, and evolving regulatory frameworks.

## Supplementary material

10.2196/93890 Multimedia Appendix 1 Center-level methodological characteristics, cross-center variations in ultrasound reporting language, standardized prompts, operational definitions of nonstandard large language model outputs, and additional diagnostic performance analyses (pairwise comparisons, tie-breaking events, sensitivity analyses, and Bayesian prevalence-adjusted estimates).

## Abbreviations

AUC: area under the curve
C-TIRADS: Chinese Thyroid Imaging Reporting and Data System
FNA: fine-needle aspiration
LLM: large language model
NPV: negative predictive value
PPV: positive predictive value
RECIST: Response Evaluation Criteria in Solid Tumors
ROC: receiver operating characteristic
