# Supplementary material for: The Performance of ChatGPT-4o and DeepSeek-R1 in Interpreting Thyroid Nodule Ultrasound Text Reports: Multicenter Study
Source: J Med Internet Res. 2026 Jul 28;28:e93890. doi: 10.2196/93890 (PMC13412018; doi:10.2196/93890)
Supplement: Multimedia Appendix 1 [file jmir-v28-e93890-s001.docx]

Supplementary Table S1. Center-level methodological characteristics

| Characteristic | Center A | Center B | Center C |
| --- | --- | --- | --- |
| Institution | The Affiliated Hospital of Qingdao University | Tai 'an City Central Hospital | JiaoZhou Central Hospital of Qingdao |
| Hospital tier | 3A (A+) | 3A (A) | 3A (recent; prior B+) |
| Ultrasound equipment | Aloka Arietta 70 (Hitachi, Japan); Siemens S2000 (Siemens, Germany) | Philips EPIQ 7 (Philips, Netherlands) | GE Voluson E10 (GE, USA) |
| Linear probe frequency (MHz) | 7.5–14.0 | 8.0-12.0 | 7.0-12.5 |
| No. of radiologists | 2 | 1 | 1 |
| Radiologist experience (years, professional rank） | 12, Chief Physician; 23, Associate Chief Physician | 29, Chief Physician | 33, Chief Physician |
| Reporting template | Structured (C-TIRADS 2020–aligned) | Structured (C-TIRADS 2020–aligned) | Structured (C-TIRADS 2020–aligned) |
| C-TIRADS version applied | 2020 | 2020 | 2020 |
| Standardized C-TIRADS training | Yes | Yes | Yes |
| No. of clinicians | 5 | 3 | 3 |
| Clinician experience (years, mean±SD [range]) | 12.5±4.2 [8–20] | 10.2±3.5 [5–16] | 9.8±3.1 [4–15] |
| Clinical management guideline followed | Guidelines for the diagnosis and management of thyroid nodules and differentiated thyroid cancer | Guidelines for the diagnosis and management of thyroid nodules and differentiated thyroid cancer | Guidelines for the diagnosis and management of thyroid nodules and differentiated thyroid cancer |

Supplementary Table S2. Examples of Cross-center Variations in Ultrasound Reporting Language for Key Thyroid Nodule Features

| Ultrasound Feature | Center A | Center B | Center C | Semantic Equivalence |
| --- | --- | --- | --- | --- |
| Echogenicity | 低回声 （low echogenicity） | 回声减低 （decreased echogenicity） | 偏低回声 （slightly low echogenicity） | Equivalent |
| Margin | 边界不清晰 （unclear boundary） | 边界欠清 （ill-defined boundary） | 边缘模糊 （blurred margin） | Equivalent |
| Calcification | 微钙化 （microcalcification) | 细小点状强回声 （tiny punctate hyperechoic foci） | 砂粒样钙化 （sand-like calcification） | Equivalent |
| Aspect ratio | 纵横比>1 （A/T ratio >1） | 前后径>左右径 （AP diameter > transverse diameter） | 竖立位生长 （taller-than-wide） | Equivalent |
| Vascularity | 内部血流丰富 （rich internal blood flow） | CDFI示较多血流信号 （CDFI shows abundant flow signals） | 血供丰富 （rich blood supply） | Equivalent |
| Shape | 形态不规则 （irregular shape） | 形态欠规则 （somewhat irregular shape） | 边缘不规整 （irregular contour） | Equivalent |
| Composition | 实性结节 （solid nodule） | 以实性为主 （predominantly solid） | 实性低回声结节 （solid hypoechoic nodule） | Equivalent |

Note: All examples are representative excerpts from de-identified reports. Despite variations in phrasing, all descriptions convey semantically equivalent clinical information as confirmed by a senior radiologist panel review.

Supplementary Table S3: Standardized Prompts Used in the Experiments (Original Chinese with English Translation)

| Prompt | Task | Original Chinese | English Translation |
| --- | --- | --- | --- |
| 1 | Benign–malignant differentiation | 你作为一名经验丰富的高级超声专家，在诊断甲状腺结节方面具有广泛的专业知识，请根据以下超声检查描述（包括结节位置、方向、边缘、晕环、成分、回声强度、回声质地、回声灶、后壁特征及大小）判定该结节的良恶性，并提供诊断依据。最后，请给出明确的诊断结论。 | As an ultrasound expert with expertise in diagnosing thyroid nodules, please determine the nodule's benign or malignant nature and provide your diagnostic rationale based on the provided ultrasound examination description, such as location, orientation, margin, halo, composition, echogenicity, echotexture, echogenic foci, posterior feature, and size. Finally, please provide a clear diagnostic conclusion. |
| 2 | C-TIRADS classification | 你作为一名经验丰富的高级超声专家，在诊断甲状腺结节方面具有广泛的专业知识，请根据以下超声检查描述，按照C-TIRADS指南判断结节风险等级(1、2、3、4a、4b、4c、5), 要求结果确切，不可存在模棱两可的答案。 | As a senior ultrasound expert with extensive professional knowledge in diagnosing thyroid nodules, please determine the malignant risk level of the nodule (1, 2, 3, 4a, 4b, 4c, 5) according to the following ultrasound examination description in accordance with the C-TIRADS guidelines. The result should be definite without ambiguous answers. |
| 3 | Management recommendation | 你作为一名甲状腺方面的资深临床专家，在诊断甲状腺结节方面具有广泛的专业知识，请根据以下超声检查描述和超声医师提示（超声医师已根据C-TIRADS指南对结节进行分类），给出最优管理意见（随诊复查/穿刺）。 | As a senior clinical expert in thyroid diseases with extensive expertise in diagnosing thyroid nodules, please offer the optimal management recommendation (follow-up/FNA) based on the provided ultrasound examination description and the radiologist's classification of the nodules according to the C-TIRADS guidelines. |

Note: The Original Chinese prompts were used in all experimental iterations for model input. English translations are provided solely for the convenience of international readers and were not used during the actual experiments. The English translations were produced by two bilingual researchers with medical backgrounds and cross-checked for semantic equivalence with the original Chinese versions.

Supplementary Table S4. Operational Definitions and Representative Examples of Non-standard Large Language Model Outputs

| Output category | Definition | Task | Representative example |
| --- | --- | --- | --- |
| Format error | Output failed to provide a response conforming to the required answer format | Benign–malignant differentiation | “根据上述超声特征，该结节具有一定的恶性风险，提示需要进一步评估”(Descriptive commentary only; no definitive benign or malignant diagnosis provided) |
|  |  | C-TIRADS classification | “根据超声特征，该结节为C-TIRADS 4类”(Category 4 reported without the required sub-classification into 4a, 4b, or 4c) |
|  |  | Management recommendation | “建议尽快外科会诊，评估手术指征”(Surgical referral recommended instead of selecting from the predefined options of follow-up or FNA) |
| Equivocal response | Output contained hedged or conditional language without committing to a single definitive answer | Benign–malignant differentiation | “该结节倾向于恶性，但不能完全排除良性可能”(Hedged conclusion without a single definitive diagnosis) |
|  |  | C-TIRADS classification | “该结节考虑C-TIRADS 4a，但部分特征符合4b标准，建议结合临床综合判断”(Multiple alternative categories provided without a single definitive classification) |
|  |  | Management recommendation | “若结节持续增大，建议行穿刺活检；若无明显变化，可继续随诊复查“(Conditional recommendation contingent on clinical scenarios rather than a single definitive management decision) |
| Hallucination | Output contained information does not present in or supported by the input data, including fabricated clinical findings, spurious reasoning steps, or non-existent guideline recommendations | — | “该低回声结节伴有点状钙化，边界不清”(Punctate calcifications cited as a diagnostic feature despite not being described in the input data) |
|  |  | — | “该结节纵横比大于1，提示垂直生长方式，符合恶性结节由包膜向外侵袭性生长的病理机制”(Aspect ratio not provided in the input; pathological mechanism of extracapsular invasive growth fabricated by the model) |
|  |  | — | “根据C-TIRADS指南,4a类结节建议立即行外科手术治疗”(No such recommendation exists in the C-TIRADS guideline) |

Note: Representative examples are presented in Chinese, consistent with the language of the prompts and model outputs; parenthetical annotations provide English translations. C-TIRADS = Chinese Thyroid Imaging Reporting and Data System; FNA = fine-needle aspiration

Supplementary Table S5. Pairwise comparison of diagnostic performance metrics between ChatGPT-4o and DeepSeek-R1 for benign–malignant differentiation (n = 306)

| Metric | ChatGPT-4o | DeepSeek-R1 | Test method | *P*-value / Δ [95% CI] |
| --- | --- | --- | --- | --- |
| Sensitivity | 0.692 | 0.879 | McNemar | *P*<.001 |
| Specificity | 0.573 | 0.508 | McNemar | *P*=0.26 |
| Accuracy | 0.644 | 0.729 | McNemar | *P*=.008 |
| PPV | 0.704 | 0.724 | BCa Bootstrap | Δ = 0.020 [-0.026, 0.066] |
| NPV | 0.559 | 0.741 | BCa Bootstrap | Δ = 0.182 [0.088, 0.271] |
| F1 Score | 0.698 | 0.794 | BCa Bootstrap | Δ = 0.096 [0.046, 0.148] |
| AUC | 0.688 | 0.718 | DeLong | *P*=0.34 |

Note: Δ represents the difference (DeepSeek-R1 − GPT-4o); PPV = positive predictive value; NPV = negative predictive value; AUC = area under the curve; BCa bootstrap = bias-corrected and accelerated bootstrap

Supplementary Table S6. Tie‑breaking events in C‑TIRADS classification

| LLM | Tied category pairs (mode1, mode2) | Selected higher category | Frequency |
| --- | --- | --- | --- |
| ChatGPT-4o | 3, 4a | 4a | 12 |
|  | 4a, 4b | 4b | 11 |
|  | 2, 3 | 3 | 6 |
|  | 4b, 4c | 4c | 6 |
|  | 4b, 5 | 5 | 4 |
|  | 4c, 5 | 5 | 4 |
|  | 2, 4a | 4a | 3 |
|  | 1, 2 | 2 | 2 |
|  | 3, 4b | 4b | 2 |
|  | 4a, 4c | 4c | 2 |
|  | 3, 5 | 5 | 1 |
|  | Total | | 53 (5.0%) |
| DeepSeek-R1 | 4b, 4c | 4c | 30 |
|  | 4a, 4b | 4b | 24 |
|  | 3, 4a | 4a | 16 |
|  | 4a, 4c | 4c | 11 |
|  | 4c, 5 | 5 | 10 |
|  | 4b, 5 | 5 | 5 |
|  | 2, 3 | 3 | 3 |
|  | 3, 4b | 4b | 3 |
|  | 2, 4b | 4b | 2 |
|  | 2, 4a | 4a | 1 |
|  | 4a, 5 | 5 | 1 |
|  | Total | | 106 (10.0%) |

Note: Total number of cases = 1,063. Tie-breaking was performed when two or more categories shared the highest frequency; the highest C-TIRADS category was selected. All observed ties were two-way; three- or higher-way ties, or five distinct outputs with no mode at all, were not encountered. Percentages in parentheses indicate the proportion of all cases. LLMs = large language models.

Supplementary Table S7. Classification distributions and agreement with senior radiologists under different tie-breaking strategies

Panel A. C-TIRADS category distributions under different tie-breaking strategies

| Category | Radiologists, n (%) | ChatGPT-4o-High, n (%) | ChatGPT-4o-Low, n (%) | DeepSeek-R1-High, n (%) | DeepSeek-R1-Low, n (%) |
| --- | --- | --- | --- | --- | --- |
| 1 | 47 (4.4) | 15 (1.4) | 17 (1.6) | 47 (4.4) | 47 (4.4) |
| 2 | 115 (10.8) | 224 (21.1) | 231 (21.7) | 191 (18.0) | 197 (18.5) |
| 3 | 278 (26.2) | 194 (18.3) | 203 (19.1) | 175 (16.5) | 191 (18.0) |
| 4a | 285 (26.8) | 316 (29.7) | 314 (29.5) | 154 (14.5) | 173 (16.3) |
| 4b | 186 (17.5) | 206 (19.4) | 203 (19.1) | 135 (12.7) | 141 (13.3) |
| 4c | 111 (10.4) | 50 (4.7) | 46 (4.3) | 223 (21.0) | 192 (18.1) |
| 5 | 41 (3.9) | 58 (5.5) | 49 (4.6) | 138 (13.0) | 122 (11.5) |

Panel B. Squared weighted kappa agreement with senior radiologists under different tie-breaking strategies

| LLM | κ_high (95% CI) | κ_low (95% CI) | Δκ (95% CI) |
| --- | --- | --- | --- |
| ChatGPT-4o | 0.688 (0.644, 0.724) | 0.690 (0.649, 0.726) | −0.002 (−0.016, 0.013) |
| DeepSeek-R1 | 0.770 (0.742, 0.796) | 0.781 (0.754, 0.807) | −0.011 (−0.019, −0.004) |

Note: High = higher-category tie-breaking strategy (primary); Low = lower-category alternative. Total n = 1,063. Δκ = κ_high − κ_low; 95% CIs estimated by BCa bootstrap with 5,000 resamples. LLMs = large language models; CI = confidence interval.

Supplementary Table S8. Distribution of Non-standard Outputs by Category and Task

| Output category | Task (n per model) | ChatGPT-4o, n/N (%) | DeepSeek-R1, n/N (%) |
| --- | --- | --- | --- |
| Format errors |  |  |  |
|  | Benign–malignant differentiation (1,530) | 8/1,530 (0.52) | 3/1,530 (0.20) |
|  | C-TIRADS classification (5,315) | 21/5,315 (0.40) | 9/5,315 (0.17) |
|  | Management recommendation (5,315) | 14/5,315 (0.26) * | 7/5,315 (0.13) * |
|  | Overall | 43/12,160 (0.35) | 19/12,160 (0.16) |
| Equivocal responses |  |  |  |
|  | Benign–malignant differentiation (1,530) | 12/1,530 (0.78) | 5/1,530 (0.33) |
|  | C-TIRADS classification (5,315) | 18/5,315 (0.34) | 11/5,315 (0.21) |
|  | Management recommendation (5,315) | 15/5,315 (0.28) | 10/5,315 (0.19) |
|  | Overall | 45/12,160 (0.37) | 26/12,160 (0.21) |
| Hallucinations |  |  |  |
|  | Benign–malignant differentiation (1,530) | 23/1,530 (1.50) | 9/1,530 (0.59) |
|  | C-TIRADS classification (5,315) | 31/5,315 (0.58) | 14/5,315 (0.26) |
|  | Management recommendation (5,315) | 27/5,315 (0.51) | 12/5,315 (0.23) |
|  | Overall | 81/12,160 (0.67) | 35/12,160 (0.29) |
| All non-standard outputs |  | 169/12,160 (1.39) | 80/12,160 (0.66) |

Note: *Surgical referral outputs: ChatGPT-4o, 9 (64.3% of format errors); DeepSeek-R1, 6 (85.7% of format errors). N represents the total number of individual outputs per model for each task.

Supplementary Table S9. Bayesian Prevalence-Adjusted Diagnostic Performance Estimates

| Evaluator | **Sensitivity** | **Specificity** | Metric | Study Sample (Prev = 59.5%) | Adjusted (Prev = 5%) | Adjusted (Prev = 10%) |
| --- | --- | --- | --- | --- | --- | --- |
| ChatGPT-4o | 0.692 | 0.573 | PPV | 70.4% | 7.9% | 15.3% |
|  |  |  | NPV | 55.9% | 97.2% | 94.4% |
| DeepSeek-R1 | 0.879 | 0.508 | PPV | 72.4% | 8.6% | 16.6% |
|  |  |  | NPV | 74.1% | 98.8% | 97.4% |
| Senior Radiologists | 0.846 | 0.742 | PPV | 82.8% | 14.7% | 26.7% |
|  |  |  | NPV | 76.7% | 98.9% | 97.7% |

**Note: Positive and negative predictive values (PPV, NPV) are prevalence-dependent and were recalculated using Bayes’ theorem to estimate diagnostic performance at real-world thyroid malignancy prevalence rates of 5% and 10%, based on population-based studies reporting thyroid cancer prevalence of 5-10% in nodule cohorts. The following standard formulas were applied:**

$$\begin{matrix} PPV=\frac{Sensitivity\times Prevalence}{Sensitivity\times Prevalence+(1-Specificity)\times(1-Prevalence)} \end{matrix}$$

$$\begin{matrix} NPV=\frac{Specificity\times(1-Prevalence)}{Specificity\times(1-Prevalence)+(1-Sensitivity)\times Prevalence} \end{matrix}$$

Sensitivity and specificity were derived from the study sample and held constant across prevalence scenarios, as these metrics are mathematically independent of disease prevalence. The study sample prevalence was 59.5%. Point estimates are reported without confidence intervals, as the adjusted values represent hypothetical prevalence scenarios rather than observed data.
